# Use of text messages to promote medication adherence and reduce blood pressure in patients with hypertension: the ESSENCE study

**DOI:** 10.1590/0102-311XEN050023

**Published:** 2024-12-20

**Authors:** Erlan Canguçu, Priscila Ribeiro Castro, Pablo Maciel Moreira, Paola Bandeira, Kleiton Almeida, Pablo Moura Santos, Marcio Galvão Oliveira

**Affiliations:** 1 Programa de Pós-graduação em Assistência Farmacêutica - Associação de IES, Vitória da Conquista, Brasil.; 2 Instituto Multidisciplinar em Saúde, Universidade Federal da Bahia, Vitória da Conquista, Brasil.; 3 Faculdade de Farmácia, Universidade Federal da Bahia, Salvador, Brasil.

**Keywords:** mHealth, Texting, Medication Adherence, Hypertension, mSaúde, Mensagem de Texto, Adesão à Medicação, Hipertensão, mSalud, Mensaje de Texto, Cumplimiento de la Medicación, Hipertensión

## Abstract

The ESSENCE study evaluated the effect of sending text messages with and without reminders of the time of medication use on adherence to medication treatment and the reduction of blood pressure in patients with hypertension. This was a randomized, crossover, double-blind, active-controlled clinical trial, which included patients aged 30-69 years, followed up at a community pharmacy. Messages were automatically sent using a software and were received on the participants’ smartphones. Group 1 included patients who received health information via text messages regarding antihypertensive medications and hypertension control for 90 days, whereas group 2 included those who received information messages along with reminder messages at the time of each drug dose for 90 days. After a 30-day washout period, the groups were switched and received interventions for another 90 days. The 157 evaluated individuals had a mean age of 52 (±8.8) years, and most were female (76.4%). No significant difference was found in intra- and inter-group self-reported adherence in the pre- and post-crossover periods. A significant reduction was found in the pre-crossover period in both groups rather than between the groups for systolic and diastolic blood pressures. At the end of the study, group 1 had a significantly lower mean blood pressure than group 2. However, we could not differentiate which intervention was more effective in terms of outcomes, thus presenting an equivalent effect between the two interventions. These results suggest the possibility of implementing message transmission in health services.

## Resumo

O estudo ESSENCE avaliou o impacto do envio de mensagens de texto com lembretes do horário de uso dos medicamentos na adesão ao tratamento e na redução da pressão arterial em pacientes com hipertensão. Este ensaio clínico randomizado, cruzado, duplo-cego e controlado incluiu pacientes com idade de 30 a 69 anos, acompanhados em uma farmácia comunitária. As mensagens foram enviadas automaticamente por meio de um software e recebidas nos celulares dos participantes. O grupo 1 incluiu pacientes que receberam mensagens de texto informativas sobre medicamentos anti-hipertensivos e controle da hipertensão por 90 dias, enquanto o grupo 2 incluiu aqueles que receberam mensagens informativas associadas a mensagens de lembrete no horário de cada dose do medicamento por 90 dias. Após um período de *washout* de 30 dias, os grupos foram trocados e receberam intervenções por mais 90 dias. Os 157 indivíduos avaliados tinham uma média de idade de 52 (±8,8) anos, sendo a maioria do sexo feminino (76,4%). Não foi encontrada diferença significativa na adesão autorreferida intra e intergrupos antes e após o estudo. Uma redução significativa foi encontrada no período anterior ao estudo em ambos os grupos, e não entre os grupos, para as pressões arteriais sistólica e diastólica. Ao final do estudo, o grupo 1 apresentou uma pressão arterial média significativamente menor do que o grupo 2; no entanto, não foi possível diferenciar qual intervenção foi mais eficaz em termos de desfechos, apresentando um efeito equivalente entre as duas intervenções. Os resultados indicam a possibilidade de implementação de sistemas de envios de mensagens nos serviços de saúde.

## Resumen

El estudio ESSENCE evaluó el impacto del envío de mensajes de texto recordando a las personas el horario de la medicación en la adhesión al tratamiento y en la reducción de la presión arterial en pacientes con hipertensión. Este ensayo clínico aleatorizado, cruzado, doble ciego y controlado incluyó a pacientes de 30 a 69 años, en seguimiento en una farmacia comunitaria. Los mensajes se enviaron automáticamente mediante un software y se recibieron en los teléfonos móviles de los participantes. El grupo 1 incluyó a pacientes que recibieron mensajes de texto informativos sobre medicamentos antihipertensivos y control de la hipertensión durante 90 días, mientras que el grupo 2 incluyó a quienes recibieron mensajes informativos asociados a mensajes recordatorios en el horario de cada dosis de medicamento durante 90 días. Tras un período de *washout* de 30 días, los grupos se cambiaron y recibieron intervenciones durante otros 90 días. Los 157 participantes evaluados tenían una edad media de 52 años (±8,8), y la mayoría eran mujeres (76,4%). No se encontraron diferencias significativas en la adhesión autoinformada intra e intergrupos antes y después del estudio. Se constató una reducción significativa en el período anterior al estudio en ambos grupos, y no entre los grupos, para las presiones arteriales sistólica y diastólica. Al final del estudio, el grupo 1 presentó una presión arterial media significativamente menor que la del grupo 2; sin embargo, no fue posible diferenciar qué intervención fue más efectiva en términos de resultados, presentando un efecto equivalente entre ambas intervenciones. Los resultados indican la posibilidad de implementar sistemas de mensajería en los servicios de salud.

## Introduction

Systemic arterial hypertension is a serious public health issue worldwide due to being the greatest risk factor for cardiovascular disease (CVD) development ^1^. The World Health Organization (WHO) estimates that 1.2 billion people currently have hypertension worldwide. A national survey in Brazil reported a 26.3% hypertension prevalence in 2021 ^2^. Hypertension is one of the leading causes of reduced life expectancy and quality of life that results in high socioeconomic costs due to high hospitalization rates from CVD ^1^, as well as in poor outcomes in work and productivity, which can lead to loss of family income ^3^.

Hypertension treatment requires a combination of non-pharmacological interventions and drugs to control blood pressure ^1^, being easy to diagnose and showing a wide therapeutic arsenal. However, effective blood pressure control and therapeutic regimen maintenance have been difficult due to factors such as patient nonadherence to treatment ^4^. The WHO declared nonadherence to drug treatment as the greatest public health issue, particularly for chronic diseases ^5^. Adherence to drug treatment differs according to the studied population, ranging from 55% to 68.4% among patients with hypertension and those with hypertension and comorbidities, respectively ^6^.

Several interventions have been developed to improve adherence to drug treatments ^7^ ^,^ ^8^, some of which have shown positive results. However, strong evidence of consistent impact on adherence and clinical outcomes is unavailable to justify using a specific type of intervention. Moreover, the few clinical trials that examined adherence and clinical outcomes involved combinations and complex interventions ^8^. Some studies reported that using short message services (SMS) containing reminders or patient engagement messages increased patients’ adherence to medications for chronic diseases ^9^ ^,^ ^10^ ^,^ ^11^ ^,^ ^12^.

Sending text messages via cell phones is one of the most viable ways to deliver electronic reminders since it is old technology; therefore, messages can be delivered to any existing mobile device ^9^. In addition, mobile phone subscriptions are increasing and being used by different economic classes and age groups. A survey showed that over 85% of Brazilians aged ≥ 16 years reported checking incoming text messages ^13^.

Recently, text messages have been widely tested as reminders and support in various health programs ^9^, including studies or programs for improving treatment adherence, such as in hypertension ^9^ ^,^ ^10^ ^,^ ^11^ ^,^ ^12^. Although results have been promising, they must be cautiously interpreted due to the short duration of the studies. Thus, further studies should focus on which profile of text messages better improves adherence, which populations are appropriate to receive them, whether the effects of the intervention are sustainable, and which factors impact the results ^9^. Moreover, they should explore the most effective messages to promote medication adherence and blood pressure control, comparing different contents, durations, and frequencies ^11^.

Therefore, the ESSENCE study aimed to evaluate whether the impact of sending text messages with time reminders aligned with informative messages is superior to sending only informative messages on adherence to medication treatment and blood pressure in patients with hypertension.

## Methods

### Study design

This randomized, double-blind, crossover, parallel-group, active-control clinical trial included patients with hypertension from January 2021 to April 2022. Patients who visited the community pharmacy where the study was conducted were randomized into two groups with an allocation ratio of 1:1. Intervention group 1 included patients with hypertension who received general information about antihypertensive drugs (guidance on indications, formulations, drug or food interactions, response to treatment, and monitoring of adverse effects) and text messages about blood pressure care over the phone. In contrast, intervention group 2 included patients with hypertension who received the same general information about antihypertensive drugs and text messages about blood pressure care and reminder messages about the timing of each drug dose ^14^.

### Study site

The study was conducted at a community pharmacy within the municipal primary healthcare network of Vitória da Conquista, Bahia State, Brazil. The pharmacy serves patients daily from the basic health units, dispensing medications free of charge ^15^, including drugs for hypertension. In addition, pharmacists provide guidance on proper medication use and offer pharmaceutical care to patients with chronic noncommunicable diseases.

### Study participants

Inclusion criteria encompassed patients aged 30-69 years, with a confirmed diagnosis of hypertension (self-reported, by medical report, and/or with a prescription of antihypertensive drugs), outpatient use of antihypertensive drugs dispensed by the Vitória da Conquista municipal healthcare network, access to a mobile device with SMS, and ability to access and read text messages. Older individuals who could not receive or understand text messages due to cognitive impairment were excluded (*Mini-Mental State Examination* scores of 20, 25, 26.5, 28, and 29 for illiterates, people with 1-4 years of schooling, people with 5-8 years of schooling, people with 9-11 years of schooling, and people with > 11 years of schooling, respectively) ^16^. Exclusion criteria also included patients who self-reported inability to receive or understand text messages due to visual, hearing, or cognitive impairment; those with recent pregnancy or childbirth (approximately three months); breastfeeding women; and frail older adults considered incapable of using medications without assistance, assessed by a scale that measures the instrumental activities of daily living (IADL) (patients with scores below the cut-off point in the following domains: preparing and taking medications, using the telephone, mobility outside the home, and using transport) ^17^. Participants were withdrawn from the study in the event of a change in the initial drug treatment for hypertension.

### Message system

Text messages were automatically sent via an open-source system with a web management interface named MEMO (http://memo.ufba.br), which was developed for this project. Initially, all patients received messages from others to test the sending and receiving processes, which were confirmed via telephone calls after registering in the system. In total, 10% of the patients were randomly selected for weekly follow-up phone calls to confirm that SMS messages were properly received during the study period. Participants received messages free of charge and were independent of the telephone carrier.

### Intervention group 1

Group 1 received an information text message every Monday, Wednesday, and Friday during the first three months. These messages were developed in another study ^18^ using social cognitive theory to improve drug treatment adherence via behavioral changes. The cross-cultural adaptation of the messages into Brazilian Portuguese and guarantee of adequate transmission of information were conducted by two pharmacists who are fluent in English with the support of community health workers from the municipal public network.

### Intervention group 2

During the same period, group 2 received the same information messages; however, reminder messages were also sent at the time of each prescribed medication dose. These messages were sent daily, once every three days, and once a week for the first, second, and third months, respectively.

### Crossover methodology

Both groups had a 1-month washout period after the initial 90 days. During this period, any residual effects of the intervention on the outcomes assessed after their discontinuation ^19^ were excluded.

In the three months following the washout period, the groups were changed. The group that initially received only informational text messages also began to receive reminder messages at the time of each dose. Similarly, the group that initially received both information and reminder messages started to receive information messages only (once a day on Mondays, Wednesdays, and Fridays until the end of the study).

### Follow-up

Patients who met the inclusion criteria were invited to participate in the study during their pharmacy visit to receive medication. After obtaining informed consent, the first visit was conducted, in which a trained researcher conducted the initial interview to obtain patient information, including identification and sociodemographic data, habits and behaviors, history of diseases and drug treatment, objective measures, and adherence assessed using the Brazilian Portuguese version of the *Brief Medication Questionnaire* (BMQ) ^20^.

Data referring to identification, sociodemographic profiles, habits, and behaviors were obtained via documents presented by the patient or self-report. Data on medication history, including medications in use and comorbidities, were obtained from the patient’s medical prescriptions during the interview. In addition, medical reports and laboratory or imaging tests were requested. Data were collected on a computer using the KoboToolBox platform (https://www.kobotoolbox.org). The patients were randomized into one of the study groups, and the prescribed drugs were dispensed in the required amounts for 90 days.

At the second visit, which occurred 90 days after the initial visit, patients’ prescriptions were reevaluated by blinded researchers. Anthropometric, blood pressure, and adherence measurements were recorded using the BMQ. Subsequently, the prescription drugs were dispensed for a minimum of 30 days.

At the third visit, 120 days after the initial visit, prescriptions were evaluated to confirm the absence of changes related to the initially registered treatment. The same assessments were conducted on the second visit, and the prescribed drugs were dispensed for 90 days.

At the fourth visit, 210 days after the initial visit, the same procedure as the second was adopted, ending the monitoring period.

Patients who did not show up on the scheduled date were contacted by phone call and asked to participate, whereas close contacts were contacted if patients were unreachable.

### Randomization, allocation, and blinding

Block randomization was used to balance the number of participants in each group ^19^. After the initial patient data collection (first visit), an independent researcher entered the information into a database created using Microsoft Office Excel (https://products.office.com/), after which block randomization was performed.

The study patients and the team responsible for the data collection on subsequent visits to the service were blinded to the allocations made at randomization. Moreover, both sides were instructed not to discuss the content of the messages.

### Outcomes

Adherence to drug treatment was defined by the BMQ scores, and the mean systolic blood pressure (SBP) and diastolic blood pressure (DBP) were the outcomes evaluated in this study. The BMQ score classifies individuals into four categories, including adherent (no positive response), probable adherence (positive response in 1 screen), probable low adherence (positive response in 2 screens), and low adherence (positive response in ≥ 3 screens). For analysis, participants were dichotomized considering “adherent” and “probable adherence” as “adherent” and those with “probable low adherence” and “low adherence” as “non-adherent” ^21^.

Sex, age, skin color/ethnicity, schooling, alcohol consumption, smoking habit, body mass index, amount of antihypertensive medication used, amount of medication used, number of daily doses, duration of illness, and presence of comorbidities were used to characterize the profiles of the participants.

### Sample size

To estimate the sample size, data from studies that used proportions as the main outcome measure and comparisons between the two groups were considered ^22^. A sample size of 58 patients in each group was obtained considering a statistically significant difference of 25% between the groups in treatment adherence, in line with the WHO estimates, which suggest that the average adherence among patients with chronic diseases is only 50% ^5^ (assumed for intervention group 1) and 75% for group 2. This sample size also included a statistical power of 80% and an absolute precision of 5%. A percentage loss of 30% was considered to correct eventual losses, which resulted in a sample size of 78 patients in each group.

### Statistical analysis

Descriptive statistics were estimated using simple frequency measures for proportions and appropriate measures of central tendency and dispersion. The proportions of adherent and nonadherent patients were compared at baseline, during, and after the study period (at subsequent visits) in intervention groups 1 and 2 using the chi-square and McNemar tests (paired analyses).

The mean or median blood pressure and adherence values were compared according to data distribution using the Student’s t-test or Mann-Whitney test. The values in both groups were also compared at baseline and subsequent visits using the paired t-test or Wilcoxon test.

Patients who did not return on scheduled dates despite several contact attempts were considered dropouts, and their data were archived for intention-to-treat analysis. Statistical significance was set at p < 0.05, and statistical analysis was performed using the Jamovi software, version 2.2.5 (https://www.jamovi.org).

### Ethical considerations

The research was conducted following the *Resolution n. 466/2012* of the Brazilian National Health Council, which approved the guidelines and regulatory norms for research with human beings. The study procedures were in accordance with the World Medical Association Code of Ethics (*Declaration of Helsinki*) for experiments involving human subjects and the *Uniform Requirements for Manuscripts Submitted to Biomedical Journals*. This study was registered and approved by the Research Ethics Committee at the Multidisciplinary Institute of Health, Federal University of Bahia (opinion n. 3,283,725). All participants signed an informed consent form after being informed of the risks involved in the study.

## Results

**Figure 1:**
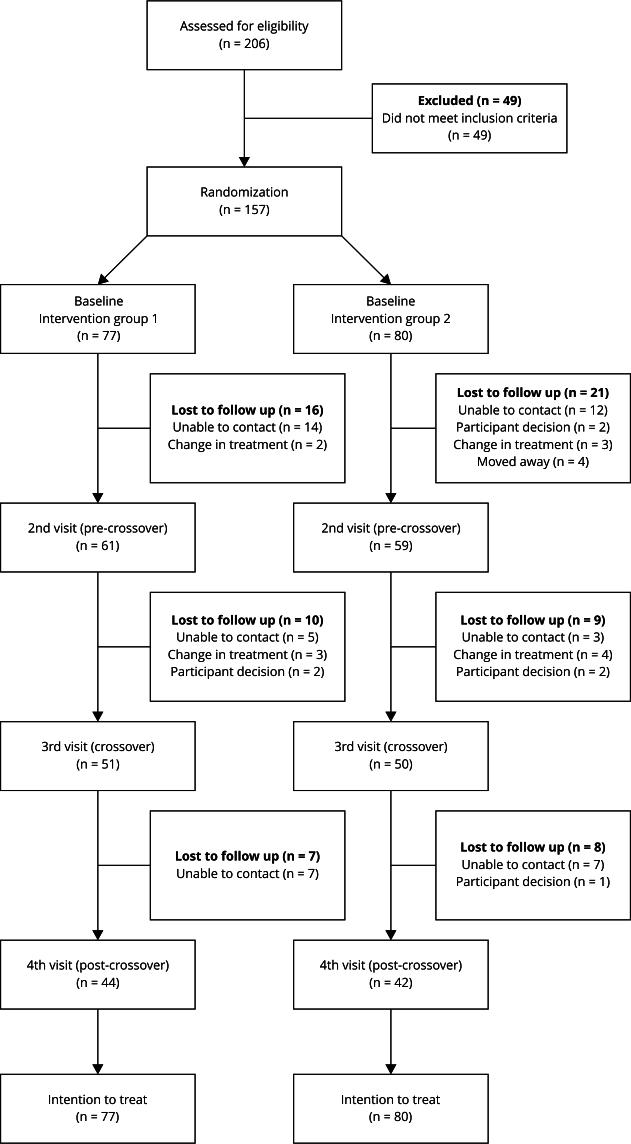
Study diagram.

Of the 206 patients evaluated for eligibility, 157 were included and randomized into groups 1, with information messages only (n = 77), and 2, with information and reminder messages (n = 80). Overall, 34 and 37 patients were lost to follow-up in groups 1 and 2, respectively (Figure 1). The final analysis was performed on 157 patients using an intention-to-treat analysis.

Among the evaluated individuals, the mean ages were 51.8 (±9.42) and 52.2 (±8.22), with 61 (79.2%) and 59 (73.8%) females in groups 1 and 2, respectively. Most participants were diagnosed with hypertension for > 5 years (64.9%) and had no other comorbidities (53.5%). Mean values of 2.78 (±1.30) and 2.66 (±1.28) drugs used and 3.56 (±1.84) and 3.55 (±1.58) daily doses were found in groups 1 and 2, respectively. The mean SBP and DBP were 134 (±18.8)/80.6 (±11.4) and 135 (±19.4)/81.6 (±9.63) for groups 1 and 2, respectively. Self-reported adherence rates were 55.8% and 65% in groups 1 and 2, respectively. No significant differences were found between the groups at baseline regarding sociodemographic data or clinical characteristics (Table 1).

**Table 1 Characteristics:** of participants at baselines.

Characteristics	Group 1 (n = 77)	Group 2 (n = 80)	p-value *
	n (%)	n (%)	
Age [mean (SD)]	51.8 (±9.42)	52.2 (±8.22)	0.77
Sex			0.41
Male	16 (20.8)	21 (26.3)	
Female	61 (79.2)	59 (73.8)	
Skin color/Ethnicity			0.83
White	16 (20.8)	14 (17.5)	
Mixed-race	40 (51.9)	45 (56.3)	
Black	21 (27.3)	21 (26.3)	
Marital status			0.17
Married or stable union	37 (48.15)	34 (42.5)	
Never been married	21 (27.3)	15 (18.8)	
Separated/Divorced	16 (20.8)	22 (27.5)	
Widower/Widow	3 (3.9)	9 (11.3)	
Schooling			0.72
Specialized program for adult education	2 (2.6)	3 (3.8)	
Complete high school	15 (19.5)	19 (23.8)	
Incomplete high school	7 (9.1)	12 (15.0)	
Elementary complete	8 (10.4)	6 (7.5)	
Incomplete fundamental	44 (57.1)	38 (47.5)	
Higher education	1 (1.3)	1 (1.3)	
Professional technician	0 (0)	1 (1.3)	
Consumption of alcoholic beverages			0.75
No	50 (64.9)	50 (62.5)	
Yes	27 (35.1)	30 (37.5)	
Smoking habit			0.23
No	68 (88.3)	75 (93.8)	
Yes	9 (11.7)	5 (6.3)	
Number of antihypertensives used [mean (SD)]	2.08 (±0.80)	2.09 (0.83)	0.94
Total number of drugs used [mean (SD)]	2.78 (±1.30)	2.66 (±1.28)	0.57
Number of daily doses [mean (SD)]	3.56 (±1.84)	3.55 (±1.58)	0.97
Diagnostic time (year)			0.09
< 1	5 (6.5)	8 (10.0)	
1-2	9 (11.7)	2 (2.5)	
2-4	17 (22.1)	14 (17.5)	
≥ 5	46 (59.7)	56 (70.0)	
Other comorbidities			0.95
No	41 (53.2)	43 (53.8)	
Yes	36 (46.8)	37 (46.3)	
BMI [mean (SD)]	29.2 (±5.18)	29.9 (±5.19)	0.36
SBP [mean (SD)]	134 (±18.8)	135 (±19.4)	0.87
DBP [mean (SD)]	80.6 (±11.4)	81.6 (±9.63)	0.54
Adherence			0.24
No	34 (44.2)	28 (35.0)	
Yes	43 (55.8)	52 (65.0)	

BMI: body mass index; DBP: diastolic blood pressure; SBP: systolic blood pressure; SD: standard deviation.

* Chi-square test/t-test.

The total adherence rate started at baseline at 60.5% and ended at 68.8% after seven months of follow-up. No significant difference was found in adherence between the groups in the pre-and post-crossover periods; however, group 2 showed better adherence in both periods than group 1. For the SBP outcome, a significant difference was found between the groups only in the post-crossover period, with lower values in group 1. Regarding DBP, a statistically significant difference was found in both periods, with lower values for group 1 (Table 2).

**Table 2:** Results of primary outcomes at baseline and subsequent visit and pre- and post-crossover.

Visit	Adherents			SBP				DBP			
	Group 1 (n = 77)	Group 2 (n = 80)	p-value	Group 1 (n = 77)	Group 2 (n = 80)	MD (95% CI)	p-value	Group 1 (n = 70)	Group 2 (n = 80)	MD (95% CI)	p-value
	n (%)	n (%)		Mean (SD)	Mean (SD)			Mean (SD)	Mean (SD)		
Baseline	43 (55.8)	52 (65.0)	0.240 *	134 (±18.8)	135 (±19.4)	-0.5 (-6.0; 6.0)	0.992 **	80.0 (±11.4)	81.6 (±9.6)	-1.0 (-4.0; 3.0)	0.587 **
2nd	47 (61.0)	56 (70.0)	0.237 *	131 (±16.5)	129 (±14.6)	2.0 (-2.0; 4.0)	0.226 **	77.2 (±9.4)	79.0 (±8.6)	-2.0 (-4.0; -1.0)	0.017 **
3rd	50 (64.9)	56 (70.0)	0.498 *	126 (±12.7)	130 (±15.3)	-4.0 (-4.0; 1.0)	0.019 **	77.1 (±8.5)	79.2 (±8.1)	-2.0(-3.5; -1.5)	< 0.001 **
4th	51 (66.7)	57 (71.3)	0.157 *	125 (±12.0)	132 (±11.4)	-7.0 (-7.0; -7.0)	< 0.001 **	75.0 (±7.7)	81.2 (±6.1)	-6.0 (-6.0; -6.0)	< 0.001 **

95%CI: 95% confidence interval; DBP: diastolic blood pressure; MD: mean difference; SBP: systolic blood pressure; SD: standard deviation.

* Chi-square test;

** Mann-Whitney test.

No statistical difference in adherence was observed in the intragroup analyses during the two study periods. For group 1, SBP and DBP significantly decreased in both periods, although a greater reduction occurred in the pre-crossover period. However, for group 2, a greater decrease in SBP and DBP was observed in the pre-crossover period, which increased in the post-crossover period (Table 3).

**Table 3:** Intragroup paired analysis in pre- and post-crossover periods.

	1st visit (baseline)	2nd visit (pre-crossover)		p-value	3rd visit (crossover)	4th visit (post-crossover)		p-value
	n (%)	n (%)			n (%)	n (%)		
Group 1 adherents	43 (55.8)	47 (61.0)		0.493 *	50 (64.9)	51 (66.7)		0.819 *
Group 2 adherents	52 (65.0)	56 (70.0)		0.505 *	56 (70.0)	57 (71.3)		0.782 *
	mmHg	mmHg	MD (95%CI)	p-value	mmHg	mmHg	MD (95%CI)	p-value
	Mean (SD)	Mean (SD)			Mean (SD)	Mean (SD)		
Group 1								
SBP	134 (±18.8)	131 (±16.5)	-3.52 (-0.07; -6.96)	0.045 **	126 (±12.7)	125 (±12.0)	-1.00 (1.00; -2.25)	0.309 ***
DBP	80.0 (±11.4)	77.2 (±9.4)	-3.40 (-1.09; -5.72)	0.004 **	77.1 (±8.5)	75.0 (±7.7)	-2.00 (-0.50; -3.00)	0.016 ***
Group 2								
SBP	135 (±19.4)	129 (±14.6)	-5.65 (-2.20; -9.10)	0.002 **	130 (±15.3)	132 (±11.4)	2.00 (4.50; 2.00)	0.005 ***
DBP	81.6 (±9.6)	79.0 (±8.6)	-3.0 (-1.00; -4.50)	0.003 ***	79.2 (±8.1)	81.2 (±6.1)	2.00 (2.00; 0.75)	< 0.001 ***

95%CI: 95% confidence interval; DBP: diastolic blood pressure; MD: mean difference; SBP: systolic blood pressure; SD: standard deviation.

* McNemar test;

** Paired t-test;

*** Wilcoxon W test.

**Figure 2:**
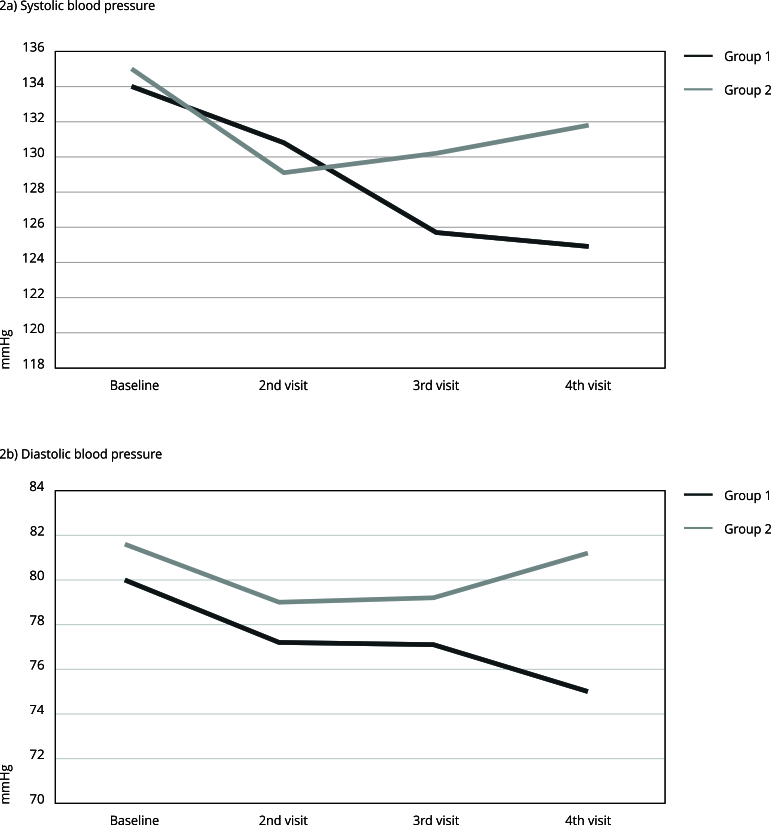
Evolution of the blood pressure means of groups in all periods.

A greater reduction in SBP in group 2 was observed in the first phase of the study, with an increase in the mean SBP from the washout period to the end of the study. In group 1, this reduction in mean blood pressure occurred steadily from baseline and was significantly lower than that in group 2 at the end of the study (Figure 2).

## Discussion

Our study evaluated the effectiveness of two types of unidirectional SMS on medication adherence and blood pressure. Regarding adherence, there was an increase in the number of adherent patients in both groups with clinical significance in both study periods. Although no statistically significant difference was found in adherence between the groups, group 2 showed greater adherence at the end of the study, which could be because this group started the study with a higher adherence rate. However, group 1 showed greater amplitude of the intervention’s impact on adherence. Thus, we cannot define the superiority of adding messages with reminders of medication use times to the information messages. Another study that evaluated the use of messages in treatment adherence also showed no difference in the self-reported adherence of individuals between those who received information messages, those who received information and reminder messages, and those who did not receive messages ^23^.

No significant differences were found in SBP and DBP between the groups during the pre-crossover period. However, at the end of the study, group 1 showed a significantly lower mean blood pressure than group 2. This suggests that individuals who received information messages and reminders in the second phase developed more consistent changes in their habits. However, we could not determine which intervention was more effective in improving the blood pressure outcome.

Intragroup analysis revealed a significant reduction in blood pressure in both groups during the study’s first phase. The decrease in mean blood pressure also has clinical significance, as studies have indicated that a 5mmHg reduction in SBP is associated with an approximately 10% decrease in cardiovascular risk. This means a reduction in the risk of multiple clinically relevant morbidities that include acute myocardial infarction (fatal or not) or new coronary heart disease, stroke (fatal or not), or hospitalization or death due to heart failure ^24^. In the post-crossover period, no significant reduction in SBP was observed in either group. This stabilization in blood pressure reduction was expected since the number of individuals moving from nonadherent to adherent conditions increased. For individuals who adhered to treatment, a greater reduction in blood pressure was not expected without the addition of antihypertensive drugs.

Notably, this effect of text messages on blood pressure corroborates the findings of a previous study conducted in South Africa that evaluated sending text messages to patients with hypertension and found a reduction in blood pressure in the first six months and a stabilization in the average blood pressure at the end of 12 months ^25^. A significant reduction in blood pressure was also found in a study conducted in China that evaluated the effectiveness of information messages and personal consultations in blood pressure control compared with placebo ^26^.

We found a greater impact of the interventions on blood pressure outcome than on adherence to treatment, and this can be attributed to two factors. First, the impact of messages contributes to behavioral changes in lifestyle, such as healthy eating and physical activity. Consequently, these behavioral changes resulted in a reduction in blood pressure, both in adherent and nonadherent individuals following drug treatment ^18^. According to self-report, patients answering questions from specific instruments is an indirect method of assessing adherence, and its accuracy can be impaired by a memory bias, misunderstanding of the questions, or even an embarrassment in answering ^27^. The BMQ, which is both practical and flexible, is one of the most widely used instruments in research. However, it only evaluates adherence for the most recent week, in addition to overestimating adherence, as the patient may accidentally or intentionally conceal information about how drug therapy actually occurred ^20^.

### Strengths and limitations

This study used simple, low-cost technology, and open-source software with a web management interface, which can be implemented in any health service. In addition, our design allowed for follow-up even after the pause and changes in the receipt of interventions, evaluating individuals at different times of the study and enabling visualization of the evolution of outcomes.

In addition to the limitations related to the assessment of adherence via self-report, conducting the study only with nonadherent patients was undecided since nonadherence is a highly changeable condition. Moreover, adherent individuals can become nonadherent within short time intervals. We also highlight that this study encountered a high number of losses due to being conducted during the COVID-19 pandemic.

## Conclusion

The ESSENCE study assessed the impact of text messages by comparing combinations of messages with different contents. Notably, the suggested interventions showed an increase in the number of adherent individuals and a significant reduction in blood pressure; thus, we found a similar impact between the two interventions for the primary outcomes evaluated. Despite this, the results indicate the effectiveness of both SMS strategies in adherence to treatment and blood pressure control, which might enable the implementation of messaging systems in health services. However, further studies should be conducted using only nonadherent individuals with uncontrolled blood pressure, in addition to using strategies with longer follow-up time and different message sending frequencies.
